# Altered Immune Phenotypes and *HLA-DQB1* Gene Variation in Multiple Sclerosis Patients Failing Interferon *β* Treatment

**DOI:** 10.3389/fimmu.2021.628375

**Published:** 2021-05-25

**Authors:** Priyanka Devi-Marulkar, Carolina Moraes-Cabe, Pascal Campagne, Béatrice Corre, Aida Meghraoui-Kheddar, Vincent Bondet, Alba Llibre, Darragh Duffy, Elisabeth Maillart, Caroline Papeix, Sandra Pellegrini, Frédérique Michel

**Affiliations:** ^1^ Cytokine Signaling Unit, Department of Immunology, Institut Pasteur, Paris, France; ^2^ INSERM U1221, Department of Immunology, Institut Pasteur, Paris, France; ^3^ Center of Bioinformatics, Biostatistics and Integrative Biology, Institut Pasteur, Paris, France; ^4^ Translational Immunology Laboratory, Department of Immunology, Institut Pasteur, Paris, France; ^5^ Department of Neurology, Pitié-Salpêtrière Hospital, Paris, France

**Keywords:** multiple sclerosis, type I interferon, T cells, interferon-stimulated genes, HLA class II genes, immune phenotypes, blood biomarkers

## Abstract

**Background:**

Interferon beta (IFN*β*) has been prescribed as a first-line disease-modifying therapy for relapsing-remitting multiple sclerosis (RRMS) for nearly three decades. However, there is still a lack of treatment response markers that correlate with the clinical outcome of patients.

**Aim:**

To determine a combination of cellular and molecular blood signatures associated with the efficacy of IFN*β* treatment using an integrated approach.

**Methods:**

The immune status of 40 RRMS patients, 15 of whom were untreated and 25 that received IFN*β*1a treatment (15 responders, 10 non-responders), was investigated by phenotyping regulatory CD4^+^ T cells and naïve/memory T cell subsets, by measurement of circulating IFN*α*/*β* proteins with digital ELISA (Simoa) and analysis of ~600 immune related genes including 159 interferon-stimulated genes (ISGs) with the Nanostring technology. The potential impact of HLA class II gene variation in treatment responsiveness was investigated by genotyping *HLA*-*DRB1, -DRB3,4,5, -DQA1*, and -*DQB1*, using as a control population the *Milieu Interieur* cohort of 1,000 French healthy donors.

**Results:**

Clinical responders and non-responders displayed similar plasma levels of IFN*β* and similar ISG profiles. However, non-responders mainly differed from other subject groups with reduced circulating naïve regulatory T cells, enhanced terminally differentiated effector memory CD4^+^ T_EMRA_ cells, and altered expression of at least six genes with immunoregulatory function. Moreover, non-responders were enriched for *HLA-DQB1* genotypes encoding DQ8 and DQ2 serotypes. Interestingly, these two serotypes are associated with type 1 diabetes and celiac disease. Overall, the immune signatures of non-responders suggest an active disease that is resistant to therapeutic IFN*β*, and in which CD4^+^ T cells, likely restricted by DQ8 and/or DQ2, exert enhanced autoreactive and bystander inflammatory activities.

## Introduction

Multiple sclerosis (MS) is an autoimmune and inflammatory disease of the central nervous system (CNS), leading to axonal demyelination, neuronal dysfunction, and neurodegeneration. These damages result from repeated attacks of several innate and adaptive immune cell types which have crossed blood–CNS barriers and exert a pathogenic activity together with resident activated microglia and macrophages (1, 2). Relapsing-remitting MS (RRMS), the most common form of the disease mainly affecting young adults with a female to male ratio of ~2.5–3 (3, 4), has been treated by type I interferon beta (IFN*β*) for nearly three decades. To date, IFN*β* and its highly stable pegylated form remain widely prescribed as a first-line disease-modifying therapy. Recent meta-analyses performed in a ‘real-world’ setting have confirmed the long-term efficacy of IFN*β* in delaying disability and disease progression, and decreasing mortality risk (5, 6). However, ~30% of patients are or become non-responsive to the treatment while still being potentially subject to side effects (7). Given the increasing number of novel and targeted therapeutic options for RRMS, including injectable or oral first-line therapies (8, 9), it is critical to identify IFN treatment response biomarkers and better understand the mechanisms of disease onset and pathogenesis.

Susceptibility to MS is under the influence of genetic heritability [~50% of overall risk (10)] as well as of environmental and lifestyle factors (11). Non-genetic factors such as gender, Epstein–Barr virus infection, smoking, low vitamin D, or adolescent obesity are considered to contribute to, or to potentially trigger, disease onset. Recent large-scale genome-wide association studies uncovered up to 233 independent genetic associations with MS, 30 of these mapping across the MHC region (10, 12, 13). Most variants are related to the adaptive immunity, with a group of HLA class II allelic risk variants, dominated by HLA DRB1*15:01 (OR~3.9) (13–15). Moreover, the interaction between the latter variant and non-genetic risk factors leads to a much higher susceptibility to develop MS (11).

Immune dysregulation is a hallmark of MS pathogenesis. Key players are CD4^+^ and CD8^+^ T cells that drive autoreactive and deleterious responses within the CNS while also promoting activation of myeloid and B cells. Notably, the association between specific HLA class II variants and MS points not only to the critical role of autoreactive CD4^+^ T cells whose T cell receptor is restricted by these variants, but also to antigen-presenting cells expressing the variants and among them B cells. In fact, B cells were reported to drive T cell autoproliferation in RRMS patients bearing the HLA-DR15 haplotype and to contribute to autoimmune and pro-inflammatory cytokine responses (16–18). Their key role in MS pathophysiology is demonstrated by the impressive therapeutic effect of anti-CD20-based treatments (19).

Autoreactive cells can be activated by CNS and non-CNS derived antigens through various mechanisms such as molecular mimicry following viral reactivation, recognition of neo-autoantigens and/or bystander activation (1, 17, 20). In addition, autoreactivity and pro-inflammatory T cell responses can be promoted by dysfunctional regulatory mechanisms, such as those involving peripheral CD4^+^ regulatory T cells (Tregs) and type 1 regulatory T cells (Tr1s) (21–23). Reduced thymic output of naïve Treg cells in RRMS patients may also indicate a defect of central tolerance mechanisms or an alteration of Treg homeostasis (24, 25). Conversely, effector CD4^+^ T helper subsets such as Th17 and Th1/Th17 are increased in the periphery, display enhanced pro-inflammatory cytokine and gene expression programs, and may be more resistant to Treg activity (26–28). Circulating cytotoxic CD28^−^ CD4^+^ T cells were also found to be expanded and to correlate with disease activity (29–31). Finally, CD8^+^ T cells are considered to play an important role in MS pathogenesis. These cells are enriched in cerebrospinal fluid (CSF) and CNS lesions and can be detected in the periphery with an activated effector/migratory phenotype (2, 32).

The therapeutic activity of IFN*β* is largely attributed to the induction of a global anti-inflammatory program although its direct antiviral and pro-apoptotic activities may also contribute (20, 33, 34). Various mechanisms of action involving almost all immune cell types have been proposed. Among the major immunomodulatory effects of IFN*β* treatment are the restoration or induction of regulatory T and B cell responses (22, 35–37), the reduced differentiation of inflammatory Th17 and B cells, and the attenuation of monocyte activation (33, 36, 38, 39). Many studies have also shown the strong promoting activity of IFN*α*/*β* on IL-10 expression (33, 40, 41).

Different findings have been reported on the frequency of circulating Treg, naïve and memory CD4^+^ and CD8^+^ T cell subsets in untreated and IFN-treated RRMS. The rapid evolution of phenotyping procedures, heterogeneous clinical features of patients, and the duration of IFN treatment may account for data variability. To date, no single blood biomarker can predict the therapeutic efficacy of IFN*β* nor disease activity (42). On this basis, we have explored the possibility that a combination of cellular and molecular blood biomarkers may prove valuable for patient stratification. We compared the immune status of IFN responders and non-responders with that of untreated RRMS patients and healthy donors. We used an integrated approach by analyzing circulating CD4^+^ and CD8^+^ T cell subsets, mRNA expression of IFN-stimulated genes (ISGs) and other immune genes, and allelic variation of HLA class II genes. Results of this exploratory study show converging immune signatures in non-responders, suggesting dysregulation of the immune response and higher disease activity in these patients.

## Materials and Methods

### MS Patients and Healthy Controls

RRMS patients of Caucasian ethnicity were diagnosed according to the 2010 McDonald criteria (43) and were recruited at the hospital Pitié-Salpêtrière, Paris. Untreated patients did not receive any immunomodulatory or immunosuppressive treatment at least 3 months prior to blood collection. Patients treated with IFN*β*1a Avonex (30 µg, IM, once a week) were considered as non-responders if they experienced one or more relapses during the last year of treatment. Blood was collected at least two days after IFN administration on lithium heparin-tubes for flow cytometry and gene expression assays and on EDTA-tubes for genomic DNA extraction and plasma cytokine analysis. Exclusion criteria were disease activity, steroidal anti-inflammatory or immunosuppressive drugs, antibiotics, acute or chronic infectious diseases, autoimmune and inflammatory diseases other than MS, and cancer. The study was approved by the CPP 2014/17NICB and the CNIL MMS/CWR/AR1411558. Healthy controls were from the CoSImmGEn cohort of the ICAReB platform (Clinical Investigation and Access to BioResources, Institut Pasteur) and EFS (Etablissement Français du Sang, Paris). For HLA class II genotyping, controls were from the *Milieu Interieur* (MI) cohort composed of 1,000 healthy donors, French citizens with metropolitan French origin for three generations (https://clinicaltrials.gov; NCT01699893 and NCT03905993, ANR-10-LABX-69-01). MI healthy controls and RRMS patients provided written informed consent including genetic analyses. Clinical and demographic characteristics of participants are shown in **Table 1**.

**Table 1:** Clinical and demographic characteristics of RRMS patients and healthy controls.

	RRMS patient groups			Control
	Untreated	IFN responders	IFN non-responders	Healthy donors
Number	15	15	10	14
Female sex (%)	73	86	80	64
Age (years)	40 (29–55)	41 (19–57)	34 (19–55)	37.5 (22–53)
Disease duration (years)	11 (2–24)	12 (2–22)	7.5 (2–25)	–
EDSS	1 (0–3)	0 (0–2.5) *^a^	1.8 (0–4) *^b^	–
Treatment duration (years)	–	7 (2–13)	2.5 (0.7–14)	

Values shown as median and (range).

^a^Responders vs Untreated, ^b^Non-responders vs Responders. *Mann–Whitney test, p < 0.05.

### Flow Cytometry

Blood (200 µl, sampling <6 h) was washed with PBS at 1,500 rpm, 5 min, room temperature (RT). Cell pellet was incubated with antibodies premix for 20 min at RT then with viability dye (500 µl, 1/1,000) at 4°C for 30 min (eF506, eBioscience). After washing cells with cold PBS, red cells were lysed and leukocytes were fixed in 2 ml of lysis buffer (BD biosciences) for 15 min at RT in the dark. Stained cells were acquired in 200 µl PBS using MACSQuant^®^ Analyzer 10 (Miltenyi Biotech). CD4^+^ and CD8^+^ T cell subsets were analyzed using FlowJo™10 by gating on CD3^+^ cells after exclusion of dead cells and doublets. The following antibodies were used in two eight-color panels: anti-CD3-Vioblue (BW264/5), anti-CD4-APC-vio770 (VIT4), anti-CD45RA-FITC (T6D1, anti-CD8b-PE-Cy7 (SIDI8BEE), anti-CD25-PerCPeF710 (CD25-4E3), anti-CD27-PerCPvio700 (M-T271), anti-HLADR-PE (clone AC122), anti-CD127-APC (MB15-18C9) (Miltenyi Biotec, eBioscience). Treg, CD4^+^ and CD8^+^ T cell subsets were gated using the appropriate FMO control.

### IFN*α*/*β* Measurement

Plasma was obtained by centrifugation of blood at 1,500 rpm, 5 min, RT, and frozen at −80°C. IFN*α* and IFN*β* plasma levels were measured in duplicate by single molecule array (Simoa, Quanterix) digital ELISA using homebrew assays in which capture and detection monoclonal antibodies were 8H1 and 12H5 (Immunoqure AG) for IFN*α* (41), and 710322-9 and 710323-9 IgG1 for IFN*β* (PBL Assay Science) (44).

### mRNA Gene Expression

Prewarmed blood (1 ml) was incubated into TruCulture tubes (Myriad RBM) under a final volume of 3 ml, at 37°C, for 22 h. Trizol LS (Qiagen)-lyzed cell pellets were thawed on ice at least 1 h, vortexed twice at 2,250 rpm for 5 min and centrifuged at 3,500 g for 5 min at 4°C. Total RNA was extracted using nucleospin miRNA kit (Macherey-Nagel), eluted in 30 µl RNase-free water and aliquots were stored at −80°C. RNA quality was measured with NanoDrop™2000 (ThermoFisher) and the Agilent 2100 bioanalyzer (RNA 6000 Nano kit). mRNAs were quantified using the Nanostring (nCounter) technology. For that, RNA (100 ng, 20 ng/µl) was hybridized on 12-sample strips at 65°C for 16 h using the Human immunology_V2 (579 genes) codeset and a custom 9 gene codeset *(ADAR1, HERC5, ISG15, IRF2, IRF9, RIG-I, HLA-DQA1, HLA-DQB1, HLA-DRB4*). RNA/probe complexes were immobilized on a cartridge with the ‘Prep station’ and quantified by the ‘nCounter system’ within 555 fields of views. Data were normalized to internal positive and negative controls. *TBP, POLR2A, SDHA, G6PD* housekeeping genes were determined with the algorithm ‘gNorm’ (nSolver software V4). Data were analyzed using a background threshold of 15 counts and were log2-transformed for Qlucore Omics Explorer analysis (V3.4). Geomean scores of ISG were determined for each patient according to (45) after normalization of mRNA counts from human_V2 and custom codesets.

### HLA Class II Genotyping

Genomic DNA was extracted from 2 ml EDTA-blood of RRMS patients using the Nucleon BACC3 kit (GE-Healthcare) according to the manufacturer’s instructions. Briefly, blood cells were lysed at RT, and pellet was stored at −80°C. Precipitated DNA was airdried for 10 min, resuspended in DNAse/RNAse free water overnight at 4°C and quantified using Qubit dsDNA HS Assay Kit and Qubit 4 Fluorometer (Invitrogen). Class II *HLA-DQA1*, *DQB1*, *DRB1*, and *DRB3/4/5* genotypes were determined by single molecule real time sequencing of exons 2–6 and introns 2–5 at 8× resolution (Histogenetics, USA). Genotypes were converted to serotypes according to the 2010 nomenclature of HLA system (46). *HLA-DRB1, -DQA1* and -*DQB1* typing data of 1,000 healthy donors were a resource of the *Milieu Interieur* consortium. Alleles were imputed at four-digit resolution from the analysis of 5,699,237 SNPs using SNP2HLA v1.0370 (47).

### Statistical Analyses

One-way ANOVA Kruskal–Wallis with Dunn’s correction for multiple comparisons or Mann–Whitney test was utilized for scatter bar plots showing flow cytometry, gene expression and IFN*α*/*β* level data (GraphPAD Prism 8). ANOVA multigroup comparison F-test was used for analysis of gene expression shown as heatmaps (Qlucore Omics Explorer V3.4). The distribution of HLA binding and non-binding probes (Nanostring assays) was analyzed using a generalized linear model of the binomial family followed by pairwise comparisons among groups using Tukey-like correction. HLA typing data were analyzed by pairwise comparisons of frequencies between the *Milieu Interieur* data set (n = 1,000 healthy donors) and other groups using a Fisher’s test. P-values were adjusted to account for multiple testing (Center of Bioinformatics, Biostatistics and Integrative Biology (C3BI, Institut Pasteur).

## Results

### Peripheral Blood T Cell Phenotypes in IFN*β*-Treated RRMS Patients

We investigated cellular and molecular immune phenotypes in IFN*β*-treated RRMS patients who were clinically defined as responders (Resp, n = 15) and non-responders (NR, n = 10). All patients received IFN*β*1a IM (Avonex, 30 µg, once a week), which minimized possible variation of the treatment response due to dose, frequency, and type of IFN*β*. Controls were untreated patients (UT, n = 15) and healthy controls (HC, n = 14) of similar age and sex ratio. All patients presented with a mild disease score (medians EDSS, 0–1.8) and were of Caucasian ethnicity (**Table 1** and *Materials and Methods*).

**Figure 1:**
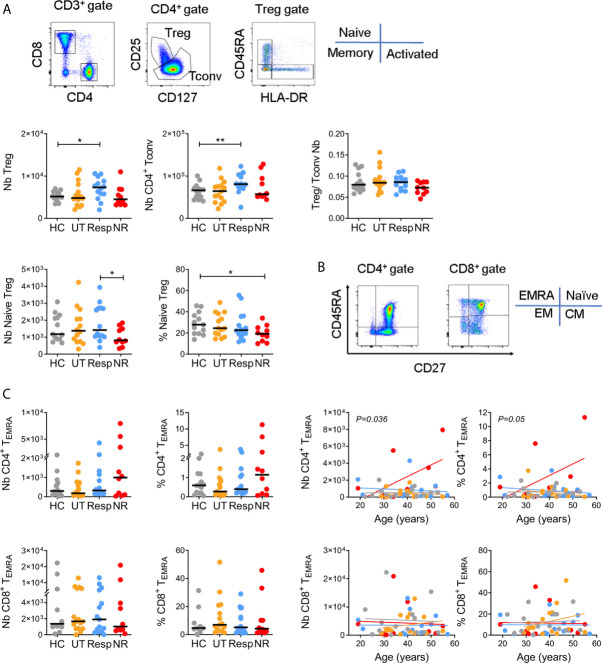
CD4^+^ T cell phenotypes in IFN responders and non-responders. **(A)** Gating strategy for CD4+ Treg and Tconv subsets. Upper panels: number (Nb) and Treg/Tconv ratios. Lower panels: number and frequency (%) of naive (CD45RA+ HLA-DR-) Tregs. Controls (HC = 13), untreated (UT = 14), responders (Resp = 14), non-responders (NR = 10). **(B)** Gating strategy for naïve (T_Naive_, CD45RA^+^ CD27^+^), central memory (T_CM_, CD45RA^−^ CD27^+^), effector memory (T_EM_, CD45RA^−^ CD27^−^), and terminally differentiated effector memory (T_EMRA_, CD45RA^+^ CD27^−^) subsets. **(C)** Left panels: number and frequency of CD4^+^ and CD8^+^ T_EMRA_. HC = 9–14, UT = 15, Resp = 14, NR = 10. Right panels: linear regressions between T_EMRA_ cells and age. Indicated *p* values were obtained from NR and Resp comparison. **(A, B)** Horizontal lines represent medians. Mann–Whitney test, *p < 0.05, **p < 0.005.

Number and frequency of regulatory T cells (Tregs), conventional CD4^+^ T cells (Tconvs), and naïve/memory CD4^+^ and CD8^+^ T cell subsets were monitored in whole blood by flow cytometry in the four subject groups (**Figures 1A, B** for gating strategies and **Supplementary Tables 1**, **2**). Based on differential expression of CD25 and CD127, we found that IFN responders displayed a significantly higher number of Treg and Tconv cells as compared to healthy donors (p < 0.05 and p < 0.005, respectively) with unchanged Treg/Tconv ratio (**Figure 1A**). In line with this, responders showed an increased number and frequency of total CD4^+^ T cells while the frequency of CD8^+^ T cells tended to be decreased in all patient groups (**Supplementary Figure 1**). Additional gating on Treg cells using CD45RA and HLA-DR identified naïve, memory and activated/terminally differentiated subsets, respectively equipped with enhanced suppressive activity potential (48). Non-responders showed a significant reduction in the number and frequency of naïve Tregs as compared with responders or healthy controls (p < 0.05) and decreased Treg/Tconv ratio (**Figure 1A**), while responders showed a trend towards increased number and frequency of memory and activated Treg subsets (**Supplementary Figure 2D**). Finally, untreated patients tended to have decreased number and frequency of activated Tregs (**Supplementary Figure 2D**).

Naïve/memory CD4^+^ and CD8^+^ T cells were analyzed based on differential expression of CD45RA and CD27 (**Figure 1B**). Alterations in numbers and frequencies of naïve, central (CM) and effector memory (EM) T cell subsets were moderate among subject groups (**Supplementary Figures 2A, B** and **Supplementary Tables 1**, **2**). However and notably, non-responders displayed a higher number and frequency of terminally differentiated effector memory cells (CD4^+^ T_EMRA_) than other subject groups, in particular with age as compared to responders (p < 0.05 for cell number, **Figure 1C**). This was not the case for CD8^+^ T_EMRA_, which suggests a selective accumulation of CD4^+^ T_EMRA_ cells in non-responders. Of note, given the strong association between CD4^+^ T_EMRA_ cells and CMV seroprevalence in healthy donors (47), we measured CMV-specific IgG in non-responders and responders but found no significant difference between the two groups (not shown).

Altogether, alterations of circulating T cell subsets were mainly observed in non-responders within the Treg and CD4^+^ T_EMRA_ compartments although we have to point out that differences were statistically significant only without correcting for multiple comparisons between subject groups.

### Circulating IFN*α*/*β* Proteins and ISG Expression in IFN*β*-Treated Patients

**Figure 2:**
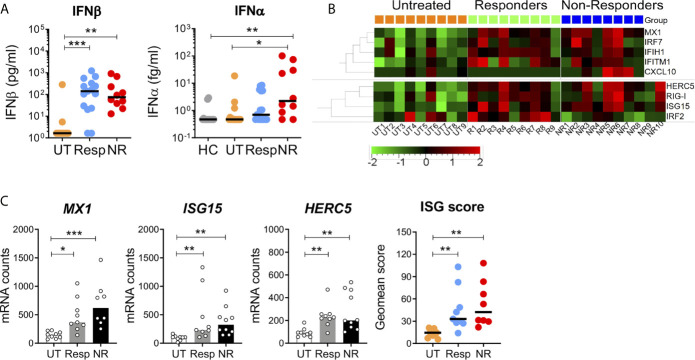
Circulating IFN*α*/*β* proteins and interferon-stimulated gene expression in blood cells. **(A)** The plasma levels of IFN*α* and IFN*β* were measured by digital ELISA (Simoa). Lower limits of detection were 0.47 fg/ml and 1.64 pg/ml for IFN*α* and IFN*β*, respectively. UT = 13–15, Resp = 15, NR = 10, HC = 14. **(B)** ISG mRNA expression in blood cells of untreated, responders and non-responders was measured with the Nanostring technology. Heatmap depicts relative mRNA counts after one-way Anova F-test, *p* < 0.05. Upper panel: human V2 codeset, UT = 9, Resp = 9, NR = 8. Lower panel: custom codeset, NR = 10. **(C)** Canonical ISGs (*MX1, ISG15, HERC5*) and geomean scores in patient groups. Kruskal–Wallis test with Dunn’s correction for multiple comparisons, *p < 0.05, **p < 0.005, ***p < 0.0005.

Next, we measured the plasma level of IFN*β* and IFN*α* proteins and ISG expression in blood cells of IFN-treated patients. We first assessed patient adherence to the treatment by measuring circulating IFN*β* using a Simoa digital ELISA. The level of IFN*β* was similar, not statistically different, between responders and non-responders (median 120 and 75 pg/ml, respectively) and was undetectable in most untreated patients (**Figure 2A**). Circulating endogenous IFN*α* was also measured using anti-pan-IFN*α* antibodies of very high affinity (49). Interestingly, non-responders exhibited a moderate but significant increase in IFN*α* (2.2 fg/ml, 0.47–101) as compared to untreated patients (0.47 fg/ml, 0.47–18.6, p < 0.05), and healthy donors (0.47 fg/ml, 0.47–2.9, p < 0.005).

ISG induction was investigated in responders and non-responders with the Nanostring digital technology that allows direct mRNA counting by probe hybridization using the human immunology V2 codeset (579 genes) and a custom codeset (nine genes). Baseline mRNA expression of 159 ISGs was compared between the two groups and untreated patients. Among these, 153 ISGs were selected from previous Nanostring data obtained with *in vitro* IFN*β*-stimulated blood of 25 healthy donors of the *Milieu Interieur* (MI) cohort (50). Analysis of ISG expression by hierarchical gene clustering and multiple comparison showed the upregulation of nine genes in IFN-treated patients (**Figure 2B**). Among these, three (*MX1*, *ISG15*, and *HERC5*) are canonical type I IFN-induced genes. Consistent with similar levels of circulating IFN*β* in responders and non-responders, the three gene ISG scores did not significantly differ in the two groups but was higher than in the untreated patients (**Figure 2C**). A different analytical strategy, based on the high fold change of ISG induction (FC > 10) in MI healthy donors, revealed a 17 gene signature in IFN-treated patients. However, a large proportion of these genes were not canonical ISGs (**Supplementary Figure 3**). Altogether, these results point to *MX1*, *ISG15*, and *HERC5* as good ISG markers for monitoring the IFN biological response in treated patients.

### Altered Gene Expression in IFN*β* Non-Responders, Including HLA Class II Genes

**Figure 3:**
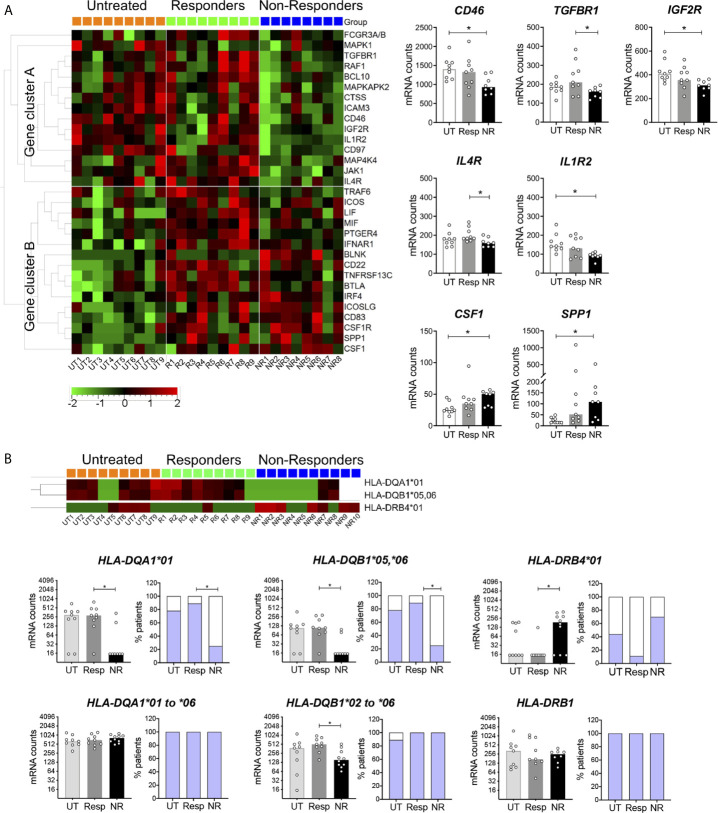
Differentially expressed genes other than ISGs in IFN non-responders. **(A)** Heatmap of mRNA expression in blood cells of patients measured with the Nanostring technology as in **Figure 2**. One-way Anova F-test, *p* < 0.05. UT = 9, Resp = 9, NR = 8–10. Right panels: genes encoding cell surface receptors and cytokine in non-responders. Kruskal–Wallis test with Dunn’s multiple comparisons, *p < 0.05. **(B)** Differential HLA-*DQA1*, *DQB1*, and *DRB4* mRNA expression in non-responders. Kruskal–Wallis test with Dunn’s multiple comparisons, *p < 0.05. UT = 9, Resp = 9, NR = 8. Two-colored bar graphs show the proportion of patients for which HLA probe binding was observed (blue) or not (white). Pairwise comparison test followed by Tukey-like adjustment for multiple comparisons, *p < 0.05.

The expression of genes other than ISGs was analyzed by hierarchical clustering in patient groups (**Figure 3A**). Non-responders differed from responders and untreated patients by 15 downregulated genes (cluster A) and two upregulated genes. Of interest, significant reductions (p < 0.05) were observed for genes encoding cell surface receptors involved in the regulation of adaptive and innate immune responses (right scatter bar graphs). For instance, CD46 and TGFBR1 control peripheral induction of Foxp3^−^ Tr1 and Foxp3^+^ Treg cells, respectively (23, 48). Interestingly, CD46-mediated induction of Tr1 cells has been reported to be altered in MS (23). IL-4R plays a crucial role in type 2 immunity, notably by promoting Th2 and B cell differentiation and by restraining neutrophil inflammatory function (51). IL-4R was also reported to dampen IL-1 response by upregulating the decoy receptor IL-1R2 (52). Consistently, a lower expression of *IL4R* and *IL1R2* was observed in non-responders. Conversely, the two cytokine encoding genes, *CSF1* and *SPP1*, were upregulated in non-responders. Interestingly, *SPP1* (*OPN*) was found to be more expressed in MS patients and to correlate with disease activity (53). Most genes contained in cluster B were upregulated in both responders and non-responders as compared to untreated patients and thus were mainly indicative of a response to the IFN treatment. Yet, a few genes were significantly more expressed in responders (**Supplementary Figure 4B**).

HLA gene expression was analyzed in patient groups, in particular classical HLA class II (*HLA-DRA, DRB1, DRB3, DRB4, DQA1, DQB1, DPA1, DPB1*) and class I (*HLA-A, B, C*) genes and non-classical class II genes (*HLA-DMA, DMB, DOB*). Based on sequence alignments, most probes were gene but not allele specific. However, *DQA1* and *DRB4* probes were preferentially directed against the *01 allele group, and the *DQB1* probe was mainly directed against the *05 and *06 allele groups. We observed in the three patient groups a binary (all or none) mode of binding of these probes (**Figure 3B**) as well as in healthy controls (not shown). Notably, non-responders strongly differed from responders by a significantly lower frequency of DQA1*01, DQB1*05,*06 probe binding and a higher frequency of DRB4*01 probe binding (p < 0.05, **Figure 3B**, upper panels). Of note, the lack of binding of both *DQA1* and *DQB1* probes was observed in the same subjects (not shown). We designed additional probes targeting all *DQA1* and *DQB1* allele groups and confirmed mRNA expression in non-responders, even if fewer *DQB1* mRNA counts were observed (**Figure 3B**, lower panels). Altogether, these data indicated differential allelic variation of *HLA-DQA1*, *DQB1*, and *DRB4* in non-responders. To further examine this possibility, IFN-treated patients were genotyped for a series of HLA class II genes.

### Increased Carriage of *HLA-DQB1* Variants Encoding DQ8 and DQ2 Serotypes in IFN*β* Non-Responders

**Figure 4:**
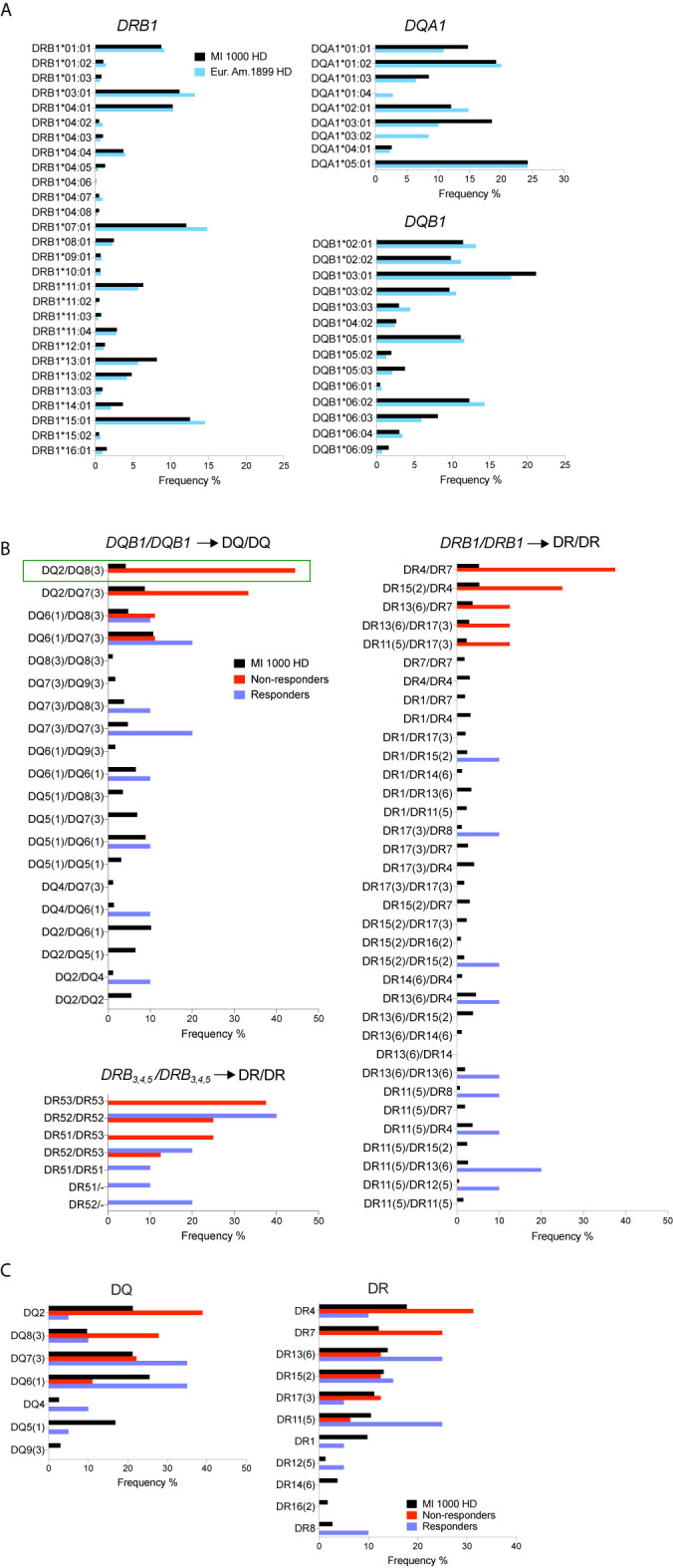
Increased carriage of *HLA-DQB1*genotypes encoding DQ2/DQ8 serotypes in non-responders. **(A)** Comparison of *HLA-DRB1, DQA1*, and *DQB1* allele frequencies in the French *Milieu Interieur* (MI) cohort (n = 1,000) and in a European-American reference cohort [n = 1,899 (54)]. **(B)** Frequencies of DQ and DR serotype pairs in MI healthy controls, IFN non-responders and responders. (Resp = 10, NR = 9 and 8 for DR53). Pairwise comparison test between groups showed statistically significant difference of the DQ2/DQ8 frequency in NR *vs* MI HD (green rectangle) with *p < 0.05, adjusted for multiple comparisons. **(C)** Frequencies of DQ and DR serotypes in MI controls, IFN non-responders and responders.

Typing of *HLA-DQA1*, *DQB1*, *DRB1*, and *DRB3,4,5* was achieved by sequencing at high resolution the available gDNA from responders (n = 10) and non-responders (n = 9). To increase statistical power, HLA typing data from the 1,000 MI healthy donors were utilized as a control reference population. First, the profile of *HLA-DQA1*, *DQB1*, and *DRB1* four-digit allelic variants, obtained by imputation from a genome-wide SNP study of the MI cohort (47), was compared to that reported in a European-American reference cohort of healthy donors [n = 1,899 (54)]. The profiles of allele frequencies were similar between the two cohorts for *DQB1* and *DRB1*, but not for *DQA1*, with the notable absence of DQA1*01:04 and DQA1*03:02 variants in the MI study (**Figure 4A**). Further analyses were focused on *DQB1*, *DRB1*, and *DRB3,4,5* that encode DQ*β* and DR*β* chains of the HLA *α*/*β* heterodimer. HLA-DQ and -DR serotypes were assigned from genotypes, and frequencies of serotype pairs were compared between responders, non-responders, and the MI 1,000 healthy controls. Strikingly, non-responders showed a marked and significant enrichment of genotypes corresponding to DQ2/DQ8 serotypes (p < 0.05, **Figure 4B**) and tended to carry *DQA1* genotypes including DQA1*03 and DQA1*02:01 variants (**Supplementary Table 3**). Non-responders were also more frequently positive for DQ2/DQ7 serotypes though this appeared to be driven by the increased usage of DQ2 and not DQ7 (**Figure 4C**). In accordance with known genetic linkage between specific HLA class II gene variants (55), non-responders carrying DR4/DR7 serotypes also carried the DR53 serotype (**Figure 4B**). As opposed to these findings, responders and MI healthy controls displayed a high diversity in the usage of DQ*β* and DR*β* chains. Overall, this genotyping analysis strongly suggests that non-responders utilize a distinct repertoire of HLA-DQ and possibly HLA-DR molecules.

## Discussion

By using an integrated approach and sensitive technologies, we have made new observations related to the therapeutic efficacy of IFN*β* in RRMS. Untreated patients and healthy controls globally showed modest differences, possibly explained by the mild MS disease score. On the other hand, non-responders and responders displayed distinct cellular and molecular immune phenotypes. Non-responders were characterized by a reduced number and frequency of naïve Tregs and higher number of CD4^+^ T_EMRA_ cells, suggesting immune dysregulation in these patients. The mechanism underlying the reduction of naïve Tregs remains to be understood. These cells may have acquired an effector-like phenotype, as for the Th1-like Treg cells described in MS patients (22). Alternatively, the decreased thymic output of naive Tregs reported in untreated patients (24, 25) or an alteration of peripheral Treg homeostasis may be more pronounced in our non-responder patients. In contrast, responders showed a higher number and frequency of total Tregs, which was mainly reflected at the level of memory and activated Treg numbers. In line with this, therapeutic IFN*β* has been proposed to promote redistribution within the Treg compartment towards memory Tregs (37, 56). Unexpectedly, we found an increase in the number of total CD4^+^ T cells in responders. This finding is consistent with studies showing that IFN*β* restores thymic function or T cell homeostasis that is altered in untreated patients (35, 57). However, other studies reported fewer circulating T cells in IFN-treated patients (37, 58). Treatment duration and time of blood collection may explain this difference. Indeed, therapeutic IFN*β* is known to induce a cytopenia depending on the dose and administration frequency (59). This effect is transient since it was observed during the first 6–12 months and resolved thereafter (60). In our study, responders were long-term treated (median 7 years) once a week, and blood was collected at least two days after IFN administration. In other studies, blood was collected earlier (<24 h) and IFN*β* was administered several times per week.

Another T cell phenotype observed in non-responders was the increase in CD4^+^ T_EMRA_ cells, in particular with age. This accumulation may be cytokine- (61) or HLA class II/antigen-driven, and it would be interesting to know whether some of these cells are autoreactive (17, 20, 30). In humans, most CD4^+^ T_EMRA_ cells are CD28^−^ (62). Interestingly, memory CD28^−^ CD4^+^ T cells with a cytotoxic and pro-inflammatory potential were reported to be clonally expanded and to associate with MS progression (29–31). This suggests that CD4^+^ T_EMRA_ cells enriched in non-responders may exert a deleterious activity.

Many studies have investigated gene expression in blood cells of IFN-treated patients, searching for a signature of IFN bioactivity and treatment response markers. However, no unified view has emerged, possibly due to variability of experimental settings (63–68). One correlate was proposed between elevated baseline ISGs, serum IFN*β* prior to treatment and poor clinical outcome (65, 67, 69). In line with this, we found that non-responders displayed an increased expression of some ISGs, in particular *MX1*, a marker previously used to study IFN non-responders (56, 70, 71). Yet, a three canonical ISG score (*MX1*, *ISG15*, *HERC5*) or other scores based on several ISGs (**Supplementary Figure 3**) did not allow for distinguishing non-responders from responders but were consistent with similar levels of circulating IFN*β* levels and IFN*β* bioactivity in the two patient groups. Among other predictive markers associated with poor IFN bioactivity and therapeutic effect is the induction of neutralizing antibodies. Of interest for our study, Avonex was reported to be the least immunogenic preparation, affecting around less than 10% patients during the first 1–3 years of treatment (70, 72–74). Thus, it is likely that a significant impact of neutralizing antibodies was not well appreciated due to the limited number of studied patients (n = 10–15/group).

The analysis of immune related genes other than ISGs led to novel observations. Non-responders were characterized by a cluster of 15 downregulated genes, including cell surface receptors (*e.g. CD46*, *TGFR1*, *IL4R*, *IL1R2*) and two upregulated cytokines (*CSF1*, *SPP1*) with an immunomodulatory function. Among these genes, *CD46* and *SPP1* have been documented to be involved in MS pathogenesis (23, 53). Together with the alteration of circulating Treg and CD4^+^ T_EMRA_ cell subsets, these results indicate some level of immune dysregulation in non-responders.

HLA allelic gene variation is known to mainly impact the antigen-binding groove formed by the *α*/*β* HLA class II heterodimer, which can result in modifications of the affinity or stability of the peptide-HLA complex and, potentially, the CD4^+^ T cell repertoire (75). Importantly, we found remarkable differences between patient groups for HLA class II gene variation. First, the binary pattern of *HLA-DQA1*, *DQB1*, and *DRB4* mRNA expression was clearly altered in non-responders as compared to untreated and responder patients. Second, the profile of HLA class II genotypes markedly differed between non-responders and 1,000 healthy donors (MI cohort).

In European populations, at least six HLA class II risk variants have been reported for MS (14, 15). The strongest one, HLA-DRB1*15:01, is part of the extended haplotype DRB5*01:01-DRB1*15:01-DQA1*01:02-DQB1*06:02 that corresponds to DR51–DR15–DQ6 serotypes. In our study, non-responders did not significantly differ from MI controls and responders for the carriage of DRB1*15:01 encoding the DR15 serotype, but they tended to be enriched for DR4/DR7 and DR53 serotypes.

The most striking result was obtained for *DQB1* in non-responders who showed a significant enrichment of DQ8/DQ2 serotypes and mainly carried *DQA1* genotypes including DQA1*03 and DQA1*02:01 allelic variants. The enrichment of DQ8 in non-responders is consistent with two recent studies (13, 76). In a meta-analysis of HLA allelic variation performed with multiple MS cohorts of European ancestry, DQB1*03:02, the single allele encoding DQ8, was identified as a dominant risk for MS. DQB1*03:02 was also found to be counteracted by the interaction with DQB1*03:01 (encoding DQ7) in this study (13). Of note, none of our non-responders carried both DQB1*03:02 and DQB1*03:01 alleles (**Supplementary Table 3**). Using next-generation sequencing, the other study associated two extended haplotypes with MS in European-American patients. The haplotype encoding DR53−DR4−DQ8 serotypes was linked with MS risk, while the other haplotype encoding DR53−DR4−DQ7 serotypes was protective (76). Hence, DQ8 and DQ7, each encoded by a single allele, appear to influence MS susceptibility in an opposite manner. In line with this, our findings support the notion that DQ8 and DQ2 may represent predictive markers of poor MS outcome in IFN-treated patients. In addition, DQ8, DQ2, and DR4−DQ8 serotypes have been strongly associated with type 1 diabetes (77) and celiac disease (78), which suggests some sharing of pathogenic mechanisms between MS and these autoimmune diseases.

Overall, our findings suggest that the disease activity in our IFN non-responder patients is such that it cannot be counteracted by IFN*β* bioactivity. Our non-responder patients may suffer from pathogenic CD4^+^ T cells, likely restricted by DQ8 and DQ2, that may exert autoreactive and bystander inflammatory activities. These findings may be of interest towards improved patient follow-up but warrant further validation with larger cohorts of patients.

## Data Availability Statement

The raw data supporting the conclusions of this article will be made available by the authors, without undue reservation.

## Ethics Statement

The study received administrative and ethical clearance in France from the “Comité de Protection des Personnes Ile de France IV (CPP)” 2014/17NICB and the “Commission Nationale de l’Informatique et des Libertés (CNIL)” MMS/CWR/AR1411558. Studies with healthy controls were approved for the CoSImmGEn cohort, ICAReB platform, Institut Pasteur (CPP 2010-dec.12483, CNIL 1161456), the Etablissement Français du Sang, Paris (CPSL UNT-18/EFS/04), and the Milieu Interieur (https://clinicaltrials.gov; NCT01699893 and NCT03905993, ANR-10-LABX-69-01). The patients provided their written informed consent to participate in this study.

## Author Contributions

PD-M performed experiments, analyzed the data, and contributed to the writing. CM-C performed experiments and analyzed the data. PC performed statistical analyses of HLA typing. BC and AM-K contributed to flow cytometry assays. VB, AL, and DD were involved in IFN measurement. CP and EM recruited patients. SP revised the manuscript. FM supervised the work and wrote the manuscript. All authors contributed to the article and approved the submitted version.

## Funding

This work was funded by the ARSEP (Aide à la Recherche sur la Sclérose En Plaques), FRM (Fondation pour la Recherche Médicale), INSERM, the Milieu Interieur ANR-10-LABX-69-01, and the Institut Pasteur, Paris.

## Conflict of Interest

The authors declare that the research was conducted in the absence of any commercial or financial relationships that could be construed as a potential conflict of interest.

## Supplementary Material

Supplementary Figure 1 Numbers and frequencies of CD3^+^, CD4^+^, and CD8^+^ T cells. Mann-Whitney test, **p* < 0.05.

Supplementary Figure 2 Numbers and frequencies of **(A)** CD4^+^ and **(B)** CD8^+^ TNaive, TCM and TEM cells in CD4^+^ and CD8^+^ CD3^+^ T cells. **(C)** Frequencies of Treg and Tconv cells in CD4^+^ CD3^+^ cells and ratio. **(D)** Numbers and frequencies of memory and activated subsets in Treg cells. Mann-Whitney test, **p* < 0.05.

Supplementary Figure 3 ISG signatures in IFN-treated patients. A list of 153 ISG (FC) > 1.5) was determined from previous MI data obtained with 25 healthy controls (50). Baseline ISG geomean scores were calculated in patient groups for strong (FC > 10, n=17 genes), moderate (FC 2.5-7.5) and low ISG induction (FC 1.5–2.5) in UT=9, Resp= 9 and NR=8. Strong ISGs (n=17) were CCL2, CCL8, CXCL10, HERC5, IFI35, IFIH1, IFIT2, IFITM1, IL1RN, IRF7, ISG15, LAMP3, MX1, RIG-I, SERPING1, TNFSF10, TNFSF13B; moderate ISG were ADAR1, BST2, CCR5, CCRL2, CDKN1A, CEACAM1, GBP1, IFI16, IRF5, IRF9, LAG3, LILRB1, MSR1, SLAMF7, SOCS1, STAT1, TAP2; and low ISGs were BLNK, CCND3, CD53, CFB, CTSC, FCER1G, HLA-C, ICAM2, LAIR1, MCL1, PLAU, PLAUR, PSMB10, PSMB8, TAPBP. Kruskal-Wallis test with Dunn’s multiple comparisons. **p* < 0.05.

Supplementary Figure 4 Expression of genes other than ISGs in IFN-treated patients. Additional genes from cluster B in **Figure 3** with **(A)** lower expression in non-responders, **(B)** higher expression in responders or non-responders. Kruskal-Wallis test with Dunn’s multiple comparisons. **p* < 0.05, ***p* < 0.005.
