# Supplementary figures and images for: Altered Immune Phenotypes and HLA-DQB1 Gene Variation in Multiple Sclerosis Patients Failing Interferon β Treatment
Source: Front Immunol. 2021 May 25;12:628375. doi: 10.3389/fimmu.2021.628375 (PMC8185344; doi:10.3389/fimmu.2021.628375)

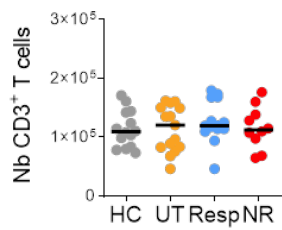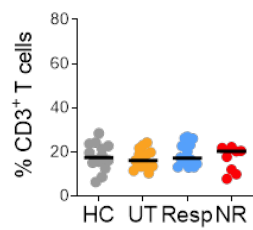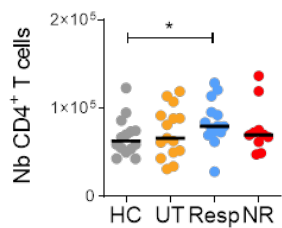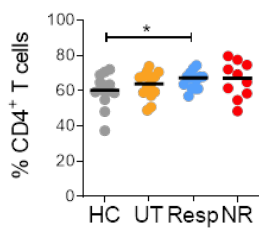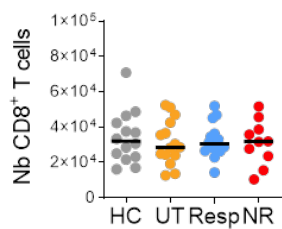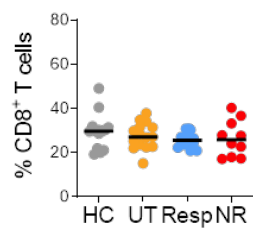

Supplement: Supplementary Figure 1 — Numbers and frequencies of CD3+, CD4+, and CD8+ T cells. Mann-Whitney test, *p < 0.05. [file Image_1.pdf]

**A** Naive/Memory CD4<sup>+</sup>

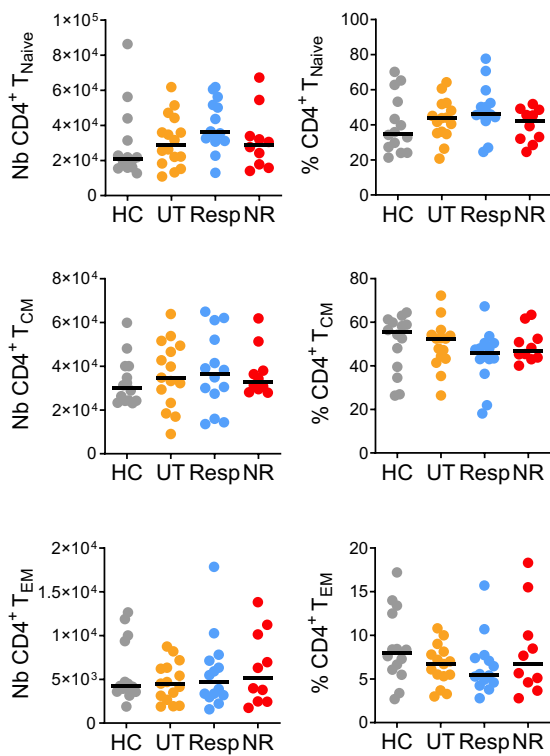

**B** Naive/Memory CD8<sup>+</sup>

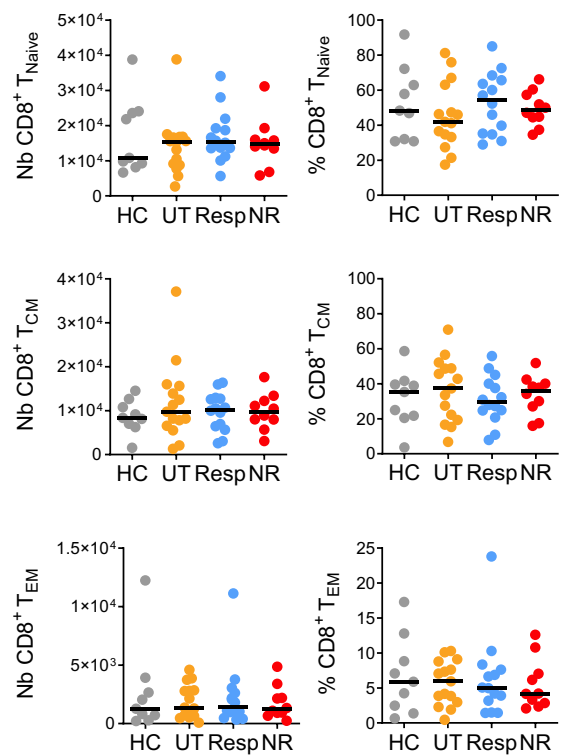

**C** Treg/Tconv

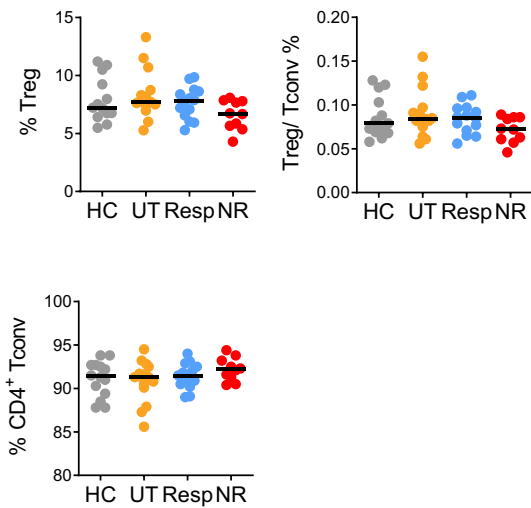

**D** Memory/Activated Treg

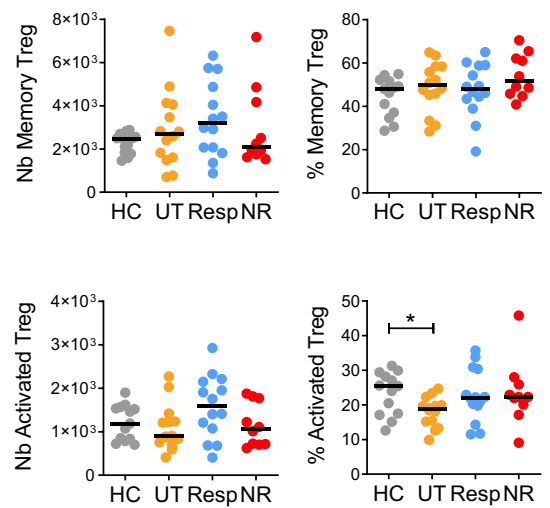

Supplement: Supplementary Figure 2 — Numbers and frequencies of (A) CD4+ and (B) CD8+ TNaive, TCM and TEM cells in CD4+ and CD8+ CD3+ T cells. (C) Frequencies of Treg and Tconv cells in CD4+ CD3+ cells and ratio. (D) Numbers and frequencies of memory and activated subsets in Treg cells. Mann-Whitney test, *p < 0.05. [file Image_2.pdf]

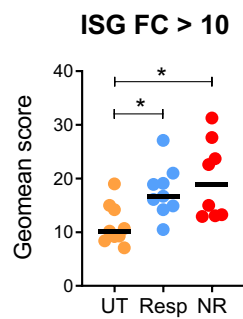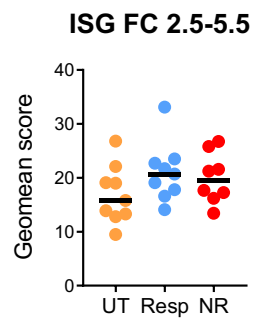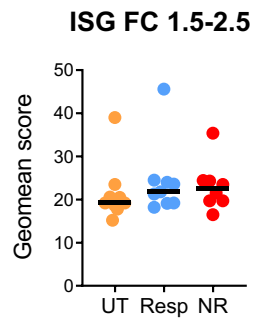

Supplement: Supplementary Figure 3 — ISG signatures in IFN-treated patients. A list of 153 ISG (FC) > 1.5) was determined from previous MI data obtained with 25 healthy controls (50). Baseline ISG geomean scores were calculated in patient groups for strong (FC > 10, n=17 genes), moderate (FC 2.5-7.5) and low ISG induction (FC 1.5–2.5) in UT=9, Resp= 9 and NR=8. Strong ISGs (n=17) were CCL2, CCL8, CXCL10, HERC5, IFI35, IFIH1, IFIT2, IFITM1, IL1RN, IRF7, ISG15, LAMP3, MX1, RIG-I, SERPING1, TNFSF10, TNFSF13B; moderate ISG were ADAR1, BST2, CCR5, CCRL2, CDKN1A, CEACAM1, GBP1, IFI16, IRF5, IRF9, LAG3, LILRB1, MSR1, SLAMF7, SOCS1, STAT1, TAP2; and low ISGs were BLNK, CCND3, CD53, CFB, CTSC, FCER1G, HLA-C, ICAM2, LAIR1, MCL1, PLAU, PLAUR, PSMB10, PSMB8, TAPBP. Kruskal-Wallis test with Dunn’s multiple comparisons. *p < 0.05. [file Image_3.pdf]

**A**

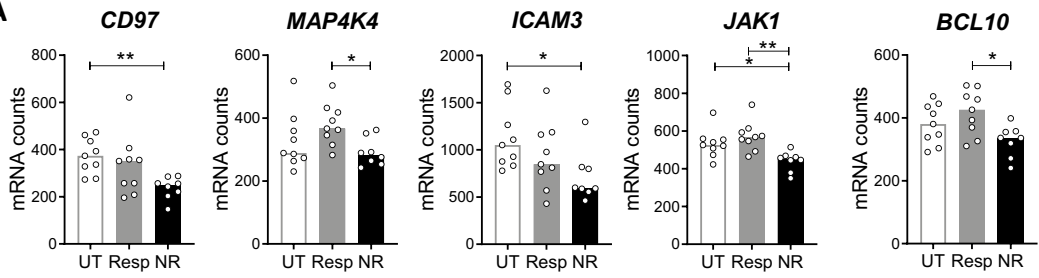

**B**

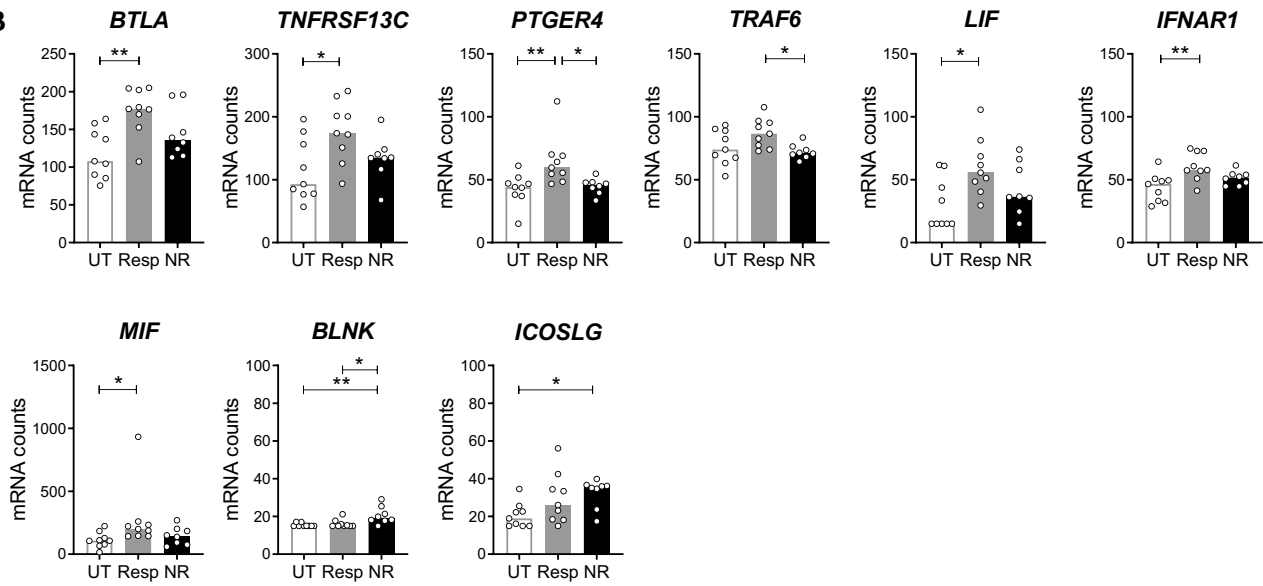

Supplement: Supplementary Figure 4 — Expression of genes other than ISGs in IFN-treated patients. Additional genes from cluster B in Figure 3 with (A) lower expression in non-responders, (B) higher expression in responders or non-responders. Kruskal-Wallis test with Dunn’s multiple comparisons. *p < 0.05, **p < 0.005. [file Image_4.pdf]
