# Supplementary material for: Altered Immune Phenotypes and HLA-DQB1 Gene Variation in Multiple Sclerosis Patients Failing Interferon β Treatment
Source: Front Immunol. 2021 May 25;12:628375. doi: 10.3389/fimmu.2021.628375 (PMC8185344; doi:10.3389/fimmu.2021.628375)
Supplement: Supplementary file 5 [file Table_1.docx]

**Supplementary TABLE 1 |** T cell subset numbers in peripheral blood of studied subjects.

| **Cell number** | | | | | | | | | |
| --- | --- | --- | --- | --- | --- | --- | --- | --- | --- |
|  | **Healthy control** | | **Untreated** | | **IFN Responder** | | **IFN Non-Responder** | |  |
|  | **Median** | **IQR** | **Median** | **IQR** | **Median** | **IQR** | **Median** | **IQR** |  |
| CD3^+^ T cells | 574 | 251 | 616 | 361 | 681 | 235 | 657 | 176 |  |
| CD4^+^ T cells | **342** | 101 | 360 | 186 | **421** | 121 | 340 | 111 |  |
| CD8^+^ T cells | 155 | 103 | 141 | 78 | 155 | 47 | 168 | 87 |  |
| Treg (CD4^+^CD25^+^CD127^-/lo^) | **26** | 7 | 24 | 17 | **37** | 14 | 23 | 15 |  |
| Tconv (CD4^+^CD25^-^CD127^+^) | **330** | 97 | 321 | 162 | **404** | 142 | 285 | 194 |  |
| Treg:Tconv ratio | 0.08 | 0.03 | 0.08 | 0.02 | 0.09 | 0.02 | 0.07 | 0.02 |  |
| ***CD4^+^ T cell subsets*** |  |  |  |  |  |  |  |  |  |
| T_naive_ (CD45RA^+^CD27^+^) | 105 | 64 | 158 | 100 | 180 | 99 | 153 | 106 |  |
| T_CM_ (CD45RA^-^CD27^+^) | 151 | 72 | 174 | 109 | 183 | 107 | 157 | 59 |  |
| T_EM_ (CD45RA^-^CD27^-^) | 21 | 23 | 23 | 20 | 23 | 19 | 20 | 26 |  |
| T_EMRA_ (CD45RA^+^CD27^-^) | 1 | 3 | 1 | 3 | 2 | 5 | 5 | 19 |  |
| ***CD8^+^ T cell subsets*** |  |  |  |  |  |  |  |  |  |
| T_naive_ (CD45RA^+^CD27^+^) | 54 | 67 | 77 | 39 | 77 | 28 | 72 | 12 |  |
| T_CM_ (CD45RA^-^CD27^+^) | 42 | 23 | 49 | 38 | 51 | 31 | 44 | 23 |  |
| T_EM_ (CD45RA^-^CD27^-^) | 6 | 13 | 7 | 11 | 7 | 8 | 5 | 6 |  |
| T_EMRA_ (CD45RA^+^CD27^-^) | 5 | 30 | 8 | 21 | 10 | 22 | 4 | 24 |  |
| ***Treg cell subsets*** |  |  |  |  |  |  |  |  |  |
| Naive Treg (CD45RA^+^HLA-DR^-^) | 6 | 6 | 7 | 6 | **7** | 8 | **4** | 4 |  |
| Memory Treg (CD45RA^-^HLA-DR^-^) | 12 | 4 | 14 | 10 | 16 | 13 | 11 | 10 |  |
| Activated Treg (CD45RA^-^HLA-DR^+^) | 6 | 4 | 4 | 3 | 8 | 5 | 5 | 5 |  |

Cell number/µl and interquartile range (IQR 75-25) counted by a MACSQuant cytometer from 200 µl blood. Numbers in bold indicate significant difference by Mann-Whitney test, p < 0.05.
