# Supplementary material for: Altered Immune Phenotypes and HLA-DQB1 Gene Variation in Multiple Sclerosis Patients Failing Interferon β Treatment
Source: Front Immunol. 2021 May 25;12:628375. doi: 10.3389/fimmu.2021.628375 (PMC8185344; doi:10.3389/fimmu.2021.628375)
Supplement: Supplementary file 6 [file Table_2.docx]

**Supplementary TABLE 2 |** T cell subset frequencies in peripheral blood of studied subjects.

| **Frequency (%)** | | | | | | | | |
| --- | --- | --- | --- | --- | --- | --- | --- | --- |
|  | **Healthy control** | | **Untreated** | | **IFN Responder** | | **IFN Non-Responder** | |
|  | **Median** | **IQR** | **Median** | **IQR** | **Median** | **IQR** | **Median** | **IQR** |
| CD3^+^ T cells | 16.7 | 7.5 | 15.7 | 5.6 | 17.6 | 8.3 | 17.7 | 9.6 |
| CD4^+^ T cells | **60.5** | 6.4 | 64.0 | 8.6 | **67.6** | 6.1 | 66.4 | 14.3 |
| CD8^+^ T cells | 30.0 | 8.0 | 27.1 | 7.7 | 25.7 | 3.4 | 25.6 | 14.1 |
| Treg (CD4^+^CD25^+^CD127^-/lo^) | 7.2 | 2.4 | 7.7 | 1.2 | 7.9 | 1.5 | 6.7 | 1.9 |
| Tconv (CD4^+^CD25^-^CD127^+^) | 91.4 | 2.1 | 91.3 | 1.8 | 91.5 | 1.8 | 92.2 | 1.70 |
| Treg:Tconv ratio | 0.08 | 0.03 | 0.08 | 0.02 | 0.09 | 0.02 | 0.07 | 0.02 |
| ***CD4^+^ T cell subsets*** |  |  |  |  |  |  |  |  |
| T_naive_ (CD45RA^+^CD27^+^) | 35.0 | 22.8 | 44.0 | 13.7 | 46.0 | 8.0 | 45.3 | 15.7 |
| T_CM_ (CD45RA^-^CD27^+^) | 55.4 | 18.0 | 50.2 | 10.0 | 45.9 | 6.7 | 45.4 | 10.9 |
| T_EM_ (CD45RA^-^CD27^-^) | 7.9 | 5.3 | 6.7 | 2.6 | 5.5 | 2.7 | 5.7 | 4.2 |
| T_EMRA_ (CD45RA^+^CD27^-^) | 0.6 | 0.9 | 0.2 | 0.6 | 0.4 | 0.8 | 0.9 | 3.9 |
| ***CD8^+^ T cell subsets*** |  |  |  |  |  |  |  |  |
| T_naive_ (CD45RA^+^CD27^+^) | 48.3 | 24.4 | 44.0 | 24.0 | 54.7 | 28.9 | 47.1 | 9.5 |
| T_CM_ (CD45RA^-^CD27^+^) | 35.3 | 18.9 | 40.1 | 25.2 | 29.4 | 14.7 | 37.7 | 10.6 |
| T_EM_ (CD45RA^-^CD27^-^) | 5.9 | 7.4 | 5.1 | 4.4 | 5.0 | 4.1 | 4.2 | 3.5 |
| T_EMRA_ (CD45RA^+^CD27^-^) | 4.6 | 15.1 | 6.4 | 11.2 | 5.2 | 14.6 | 3.7 | 13.8 |
| ***Treg cell subsets*** |  |  |  |  |  |  |  |  |
| Naive Treg (CD45RA^+^HLA-DR^-^) | **26.4** | 16.3 | 26.5 | 16.3 | 23.2 | 16.6 | **18.1** | 9.6 |
| Memory Treg (CD45RA^-^HLA-DR^-^) | 48.4 | 14.0 | 49.7 | 12.3 | 46.6 | 10.8 | 53.9 | 16.3 |
| Activated Treg (CD45RA^-^HLA-DR^+^) | **24.7** | 10.7 | **19.3** | 6.45 | 22.2 | 10.1 | 22.3 | 5.8 |

Numbers in bold indicate significant difference by Mann-Whitney test, p < 0.05.
